# Genomic Traits Associated with Virulence and Antimicrobial Resistance of Invasive Group B Streptococcus Isolates with Reduced Penicillin Susceptibility from Elderly Adults

**DOI:** 10.1128/spectrum.00568-22

**Published:** 2022-05-31

**Authors:** Shota Koide, Yukiko Nagano, Shino Takizawa, Kanae Sakaguchi, Eiji Soga, Wataru Hayashi, Mizuki Tanabe, Tomohiro Denda, Kouji Kimura, Yoshichika Arakawa, Noriyuki Nagano

**Affiliations:** a Department of Medical Sciences, Shinshu University Graduate School of Medicine, Science and Technology, Matsumoto, Japan; b Department of Laboratory Medicine, Nagano Children’s Hospital, Azumino, Japan; c Department of Health and Medical Sciences, Shinshu University Graduate School of Medicine, Matsumoto, Japan; d Nagoya University Graduate School of Medicine, Nagoya, Japan; University Paris-Saclay, AP-HP Hôpital Antoine Béclère, Service de Microbiologie, Institute for Integrative Biology of the Cell (I2BC), CEA, CNRS

**Keywords:** group B streptococci, penicillin nonsusceptible, bacteremia, elderly adults

## Abstract

This study aimed to investigate genomic traits underlying the antimicrobial resistance and virulence of multidrug-resistant (MDR) group B streptococci with reduced penicillin susceptibility (PRGBS) recovered from elderly patients with bloodstream infections, which remain poorly characterized. The pangenome was found to be open, with the predicted pan- and core genome sizes being 3,531 and 1,694 genes, respectively. Accessory and unique genes were enriched for the Clusters of Orthologous Groups (COG) categories L, Replication, recombination, and repair, and K, Transcription. All MDR PRGBS isolates retained a core virulence gene repertoire (*bibA*, *fbsA*/*-B*/*-C*, *cspA*, *cfb*, *hylB*, *scpB*, *lmb*, and the *cyl* operon), supporting an invasive ability similar to that of the other invasive GBS, penicillin-susceptible GBS (PSGBS), and noninvasive PRGBS isolates. The putative sequence type 1 (ST1)-specific AlpST-1 virulence gene was also retained among the serotype Ia/ST1 PRGBS isolates. In addition to *tet*(M) and *erm*(B), *mef*(A)-*msr*(D) elements or the high-level gentamicin resistance gene *aac(6′)-aph(2″)*, which are both rare in PSGBS, were detected among those MDR PRGBS isolates. In the core single-nucleotide polymorphism (SNP) phylogenetic tree, all invasive ST1 PRGBS isolates with serotypes Ia and III were placed together in a clade with a recombination rate of 3.97, which was 36 times higher than the value found for a clade formed by serotype V/ST1 PSGBS isolates derived mostly from human blood. ST1 has been the predominant sequence type among the PRGBS isolates in Japan, and serotypes Ia and III have been very rare among the ST1 PSGBS isolates. Thus, these lineages that mostly consisted of serotypes Ia/ST1 and III/ST1 PRGBS could possibly emerge through recombination within the ST1 populations.

**IMPORTANCE** Streptococcus agalactiae, or group B Streptococcus (GBS), is recognized as the leading cause of neonatal invasive infections. However, an increasing incidence of invasive GBS infections among nonpregnant adults, particularly the elderly and those with underlying diseases, has been observed. There is a trend toward the increasing occurrence of penicillin nonsusceptibility among GBS clinical isolates, from 4.8% in 2008 to 5.8% in 2020 in Japan. Also, in the United States, the frequency of adult invasive GBS isolates suggestive of β-lactam nonsusceptibility increased from 0.7% in 2015 to 1.0% in 2016. In adults, mortality has been significantly higher among patients with bacteremia than among those without bacteremia. Our study revealed that invasive GBS with reduced penicillin susceptibility (PRGBS) isolates harbor major virulence and resistance genes known among GBS, highlighting the need for large population-based genomic surveillance studies to better understand the clinical relevance of invasive PRGBS isolates.

## INTRODUCTION

Streptococcus agalactiae, or group B Streptococcus (GBS), is recognized as the leading cause of neonatal invasive infections like sepsis and meningitis. However, an increasing incidence of invasive GBS infections in nonpregnant adults, particularly in elderly and immunocompromised individuals with underlying diseases like diabetes mellitus, has been noted in recent years, where skin and soft tissue infections, bacteremia, osteomyelitis, and urinary tract infections are the most common clinical manifestations (1–5). In adults over 65 years of age, GBS has predominantly been associated with primary bacteremia (28 to 60%), resulting in significant mortality (6.6 to 15.6%) (1, 5, 6).

To date, 10 GBS capsular-polysaccharide-based serotypes have been described (Ia, Ib, and II to IX), and serotypes Ia, Ib, II, III, and V are most frequently associated with invasive infections (1, 2, 7, 8). In neonates, the hypervirulent clonal complex 17 (CC17) of serotype III has been responsible for late-onset GBS meningitis (9, 10). Though no association has been described between specific GBS genetic lineages and clinical presentations in adults, most invasive infections are caused by serotype V isolates, predominantly belonging to sequence type 1 (ST1), in the United States, France, and other countries (1, 7, 11–13). The recent emergence of serotype IV has been observed among adult invasive disease isolates in North America and European countries (1, 2, 4, 14). In Japan, serotype Ib/ST10 (CC10) has predominated among adults with invasive infections, followed by serotype V/ST1 (CC1), serotype III/ST19 (CC19), and serotype VI/ST1 (15–17).

GBS has a variety of virulence factors, among which the capsular polysaccharide is an important virulence determinant for invasive infection and is a target of protective immunity. Other major virulence factors include the following cell surface proteins mediating adherence and invasion: the alpha-like protein (ALP) family, comprising Alp1 (encoded by *alp1*), Alp2 (*alp2*), Alp3 (*alp3*), Alp4 (*alp4*), alpha C protein (*bca*), and Rib (*rib*); pilus island alleles PI-1, PI-2a, and PI-2b; fibrinogen-binding surface proteins A, B, and C (*fbsA*, *fbsB*, and *fbsC*); laminin-binding protein (*lmb*); C5a peptidase (*scpB*); C protein β antigen (*bac*); cell surface protease A (*cspA*); GBS immunogenic bacterial adhesin (*bibA*); hypervirulent GBS adhesin (*hvgA*); and serine-rich repeat glycoproteins (*srr1* and *srr2*) (7, 18–20). The production of various enzymes/toxins, such as β-hemolysin/cytolysin (*cyl* operon), hyaluronidase (*hylB*), and the CAMP factor pore-forming toxin (*cfb*), facilitates the bacterial entry and intracellular survival of this organism (18–20).

GBS isolates with reduced susceptibility to penicillin (PRGBS), which is the first choice for GBS disease therapy and intrapartum chemoprophylaxis, was first reported from Japan in 2008 (21, 22). Amino acid substitutions in penicillin-binding protein 2X (PBP2X) and other PBPs probably conferring reduced susceptibility to penicillin and other β-lactams have been described (21–29). Meanwhile, an increasing prevalence with a tendency to acquire a multidrug resistance phenotype to macrolides and fluoroquinolones has been noted among these PRGBS isolates (22, 23, 30). PRGBS from Japan have most commonly been recovered from respiratory specimens from elderly patients, and serotype VI/ST1 or ST458 (a single-locus variant of ST1 within CC1) was frequently associated with PRGBS isolates, including those associated with nosocomial infections (22, 23, 31). Subsequently, the occurrence of PRGBS has been described in several countries, including the United States (mostly serotype III/ST19), Canada (serotype II/ST2), and Germany (serotype Ia/ST23) (24, 25, 32). Most of them are recovered from elderly adults, while PRGBS isolates with the serotype III/ST109 (CC17) hypervirulent lineage have been identified in young infants (<90 days) with invasive infections in Mozambique (33).

Very recently, we have reported the population-level serotype replacement of serotype III by serotype Ia among ST1 multidrug-resistant (MDR) PRGBS lineages during a long-term hospital epidemic (26). Of note, a poor outcome has occurred in all patients who had MDR PRGBS isolated from blood. The clinical significance of PRGBS remains unclear, and PRGBS isolates recovered from invasive infections in adults are not well characterized at the genomic level in terms of clinical relevance. The objective of this study was to investigate the virulence and resistance gene repertoires in invasive PRGBS isolates from adults, which were compared to those in a greater number of GBS isolates.

## RESULTS

### Genomic features.

The total read counts yielded by Illumina sequencing of eight PRGBS isolates with penicillin MICs of 0.25 to 1 μg/mL that were all invasive isolates available to us (Table 1) ranged from 7,676,224 to 41,357,054, with GC contents ranging from 35.62 to 36.21%. The draft genome assemblies of these isolates contained 26 to 103 contigs/scaffolds, with total lengths of 2,248,320 to 2,355,005 bp and average GC content of 35.4%. The average nucleotide identity (ANI) values within the eight PRGBS isolates, calculated using orthologous average nucleotide identity (OrthoANI), were ≥98.02% identical. Notably, the OrthoANI values among four nosocomial-associated serotype Ia/ST1 PRGBS isolates, SU97, SU85, SU187, and SU233, ranged from 99.96 to 99.98%, and those among six PRGBS isolates comprising the four PRGBS isolates named above and two serotype III/ST1 PRGBS isolates, SF0942 and SF1510, were ≥98.43%.

**TABLE 1:** MICs of antimicrobials for eight PRGBS isolates from adults with bacteremia

Strain	Serotype	Sequence type	MIC (μg/mL) of^*a*^:															
			PEN	AMP	PIP	SAM	CTM	CTX	CAZ	CDN	IPM	MEM	GEN^*b*^	ERY	CLI	MIN	LVX	CIP^*b*^
SU85	Ia	ST1	0.25	0.5	≤2	1/0.5	4	1	8	0.5	≤0.12	0.25	16	>2	≤0.12	>8	2	2
SU97	Ia	ST1	0.25	1	≤2	0.5/0.25	4	1	16	0.5	≤0.12	0.25	8	>2	≤0.12	>8	2	2
SU187	Ia	ST1	0.25	0.5	≤2	0.5/0.25	8	1	8	0.5	≤0.12	0.25	16	>2	≤0.12	>8	>8	32
SU233	Ia	ST1	0.25	0.5	≤2	0.5/0.25	4	1	16	0.5	≤0.12	0.25	8	0.12	≤0.12	>8	>8	32
SF0942	III	ST1	0.25^*b*^	0.5	≤2	0.5/0.25	2	≤0.5	4	0.25	≤0.12	0.25	8	>2	>4	>8	>8	32
SF1510	III	ST1	0.25	0.5	≤2	0.5/0.25	2	≤0.5	8	0.25	≤0.12	0.25	8	≤0.06	≤0.12	>8	>8	32
SF0680	Ib	ST10	1	0.5	≤2	0.5/0.25	8	1	16	0.5	≤0.12	0.25	8	>2	>4	>8	>8	32
MRY08-1422	III	ST464 (CC23)^*c*^	0.25	0.5	≤2	0.5/0.25	2	≤0.5	4	≤0.12	≤0.12	0.25	>1024	0.12	≤0.12	≤0.25	>8	32

a PEN, penicillin; AMP, ampicillin; PIP, piperacillin; SAM, ampicillin-sulbactam; CTM, cefotiam; CTX, cefotaxime; CAZ, ceftazidime; CDN, cefditoren; IPM, imipenem; MEM, meropenem; GEN, gentamicin; ERY, erythromycin; CLI, clindamycin; MIN, minocycline; LVX, levofloxacin; CIP, ciprofloxacin.

b The antibiotic-containing plates were prepared in-house.

c CC, clonal complex.

### Putative ICEs carrying antimicrobial resistance and potential virulence genes.

**FIG 1:**
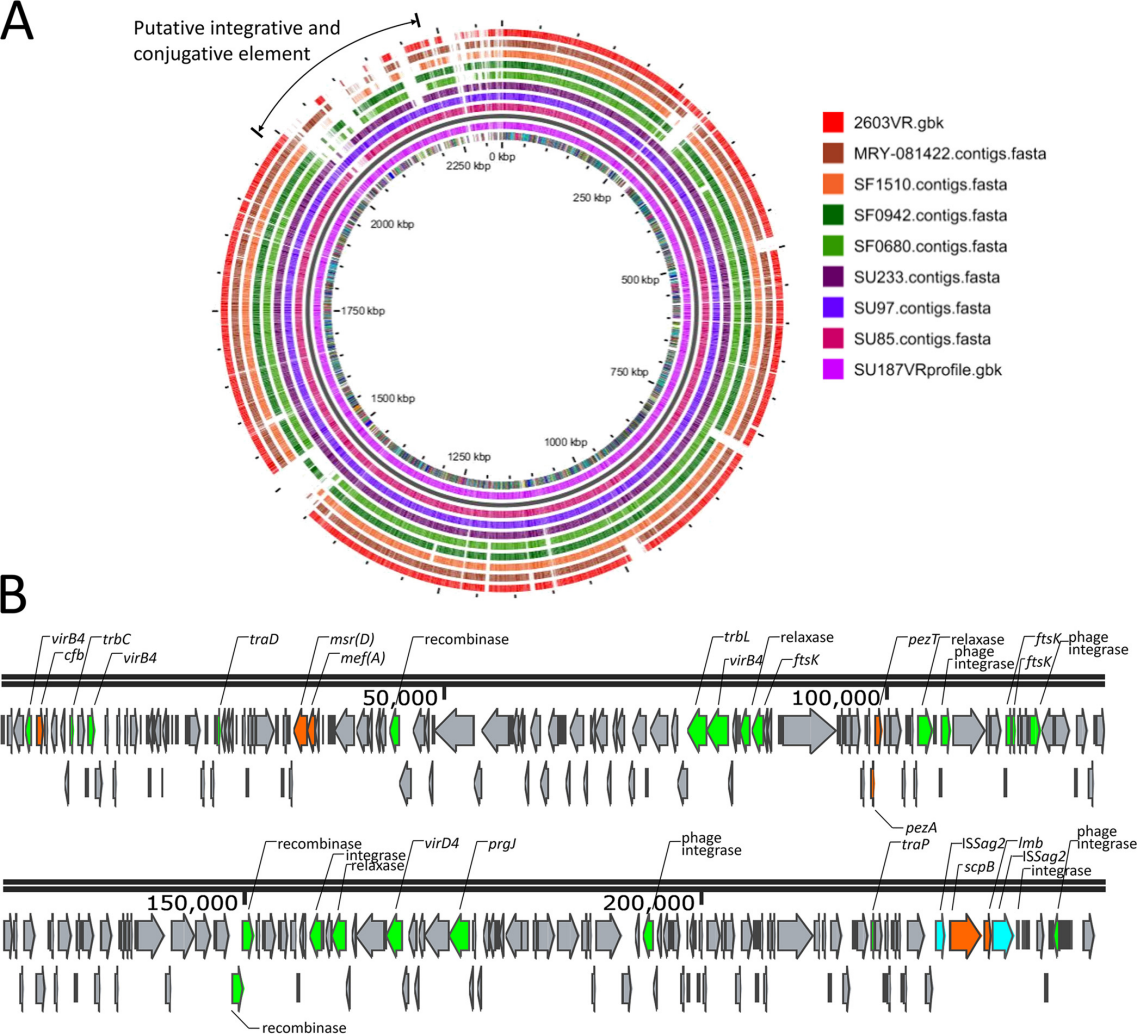
Schematic view of the putative T4SS-type integrative and conjugative element (ICE). (A) BLAST atlas of whole-genome sequences of PRGBS strains SU85, SU97, SU233, SF0680, SF0942, SF1510, MRY08-1422, and 2603V/R in comparison to the reference PRGBS strain SU187 genome. The predicted position of the T4SS-type ICE is indicated. The map was constructed using the GView server (https://server.gview.ca/). (B) Gene organization of the putative T4SS-type ICE in the PRGBS strain SU187 genome prepared by using Snapgene Viewer (https://www.snapgene.com/snapgene-viewer/). Predicted open reading frames (ORFs) are represented by arrows, with the direction of the arrow indicating transcription orientation. Genes encoding T4SS-associated mobilizable elements and mating-pair formation elements (green), virulence- and resistance-associated genes (orange), and genes with other functions or hypothetical genes (gray) are shown.

A putative integrative and conjugative element (ICE) with a type IV secretion system (T4SS-type ICEs) with a size of approximately 240 kb, which has not been described to our knowledge, was found in the draft genomes of two serotype Ia/ST1 PRGBS strains, SU97 and SU187 (Fig. 1A). The ICE, flanked by *attR* and *attL* direct repeats (TTAAAGGAATTAGCT), harbored predicted virulence genes *scpB* and *lmb*, positioned immediately downstream from *scpB*, which were surrounded by two IS*Sag2* elements, and *cfb* (Fig. 1B). The ICE also contained the antimicrobial resistance genes *mef*(A), encoding macrolide efflux major facilitator superfamily (MFS) transporter Mef(A), and *msr*(D), encoding the ABC-F-type ribosomal protection protein Msr(D), located just downstream from *mef*(A). A 5,657-bp fragment containing the *mef*(A)-*msr*(D) genes and their upstream and downstream flanking regions was 99.93 to 99.95% identical to the corresponding region in Streptococcus pneumoniae transposons, such as Tn*2009* (AF376746), Tn*2010* (AB426626), and Tn*6822* (MT489699). The presence of a sequence highly similar to this ICE was predicted in the remaining two serotype Ia/ST1 PRGBS strains, SU85 and SU233; the former contained all of the genes described above, but the latter did not contain the *mef*(A) and *msr*(D) resistance genes. The T4SS-type ICE was not predicted in the other four PRGBS isolates, serotype III/ST1 strains SF0942 and SF1510, serotype Ib/ST10 strain SF0680, and serotype III/ST464 (CC23) strain MRY-0181422, which harbored the *scpB*-*lmb* linkage and *cfb*. The presence of the Tn*916*-associated *tet*(M) was predicted in serotype Ia/ST1 strains SU85, SU97, SU187, and SU233 and serotype Ib/ST10 strain SF0680, while the *tet*(M)-*erm*(B) linkage in the Tn*916*-like Tn*3872* element (AM411624) was predicted in serotype III/ST1 strain SF0942.

### Core and pangenome structure.

**FIG 2:**
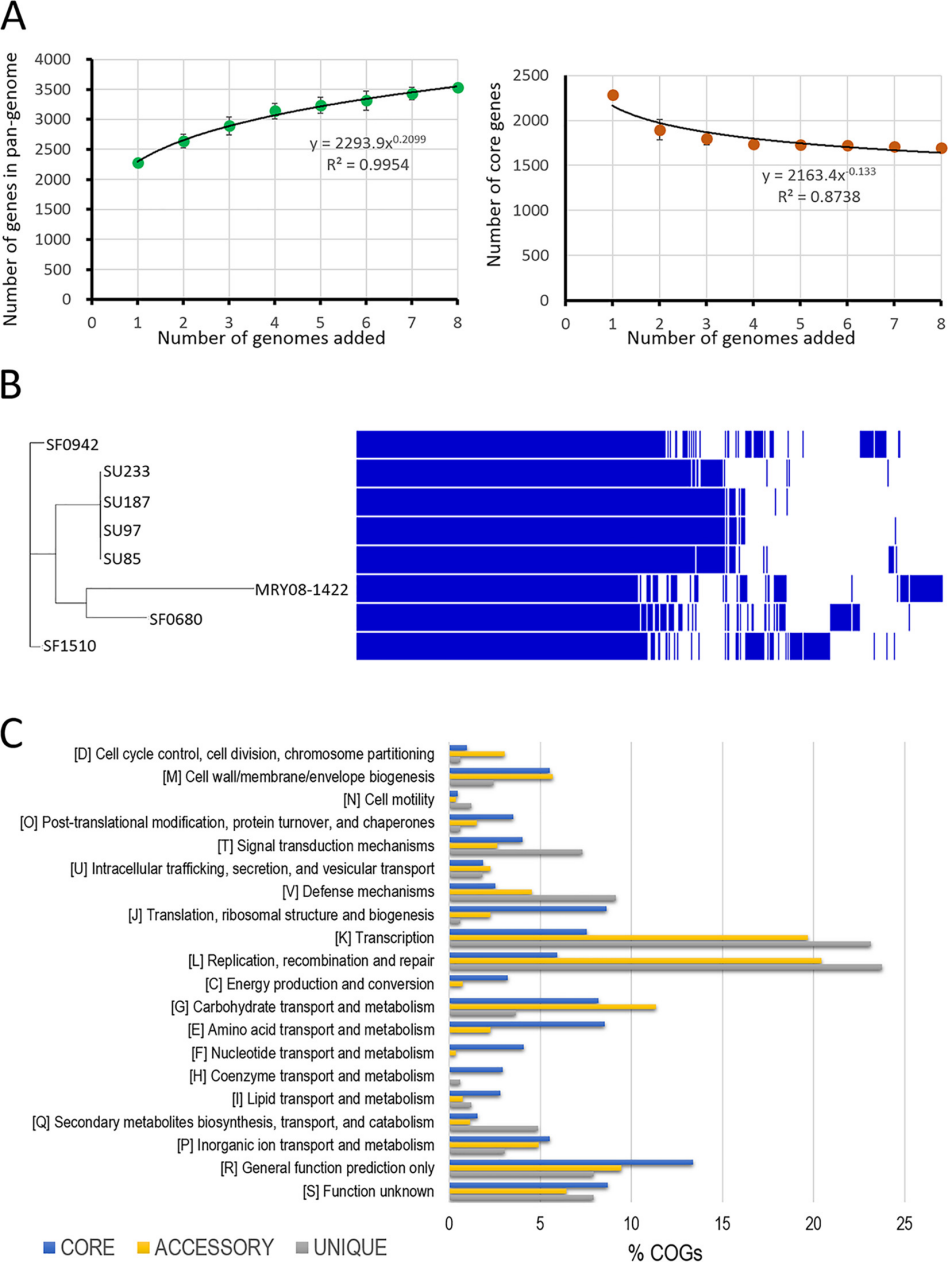
Pangenome analysis of eight invasive PRGBS isolates. (A) Pangenome (left) and core genome (right) plots for a progressively increasing number of genomes. The black line represents a power law fitting of the data. (B) Gene presence (dark blue) and absence (white) matrix in each of the eight strains. A maximum-likelihood tree of the core genome is shown on the left. (C) Distribution of COG categories in the core, accessory, and unique genomes.

Pangenome analysis of the eight invasive PRGBS isolates by Roary generated a pangenome consisting of 3,531 protein-coding genes, which was found to be 2.1 times larger than the core genome, including 1,694 genes (48.0%). Roary assigned 1,837 genes (52.0%) to the accessory genome, comprising 911 shell genes (25.8%) found in 15 to 95% of genomes and 926 cloud genes (26.2%) found in less than 15% of genomes. The pan- and core genome prediction curves yielded correlation coefficients (*r*^2^) of 0.995 and 0.874, respectively (Fig. 2A). The size of the pangenome increased continuously with the addition of every new genome, with an estimated power-law exponent of 0.210 (higher than the 0 threshold), indicating an open pangenome nature (34). Though variation in the accessory gene pool was observed within the eight PRGBS isolates, four PRGBS isolates associated with nosocomial infections, serotype Ia/ST1 strains SU85, SU97, SU187, and SU233, which were clustered together in a core genome tree, shared most of the accessory genes (Fig. 2B).

The distribution of the clusters of orthologous groups (COGs) of proteins in core, accessory, and unique genes is shown in Fig. 2C. The most abundant COG functional categories of core genes were R, General function prediction only (13.4%), followed by J, Translation, ribosomal structure, and biogenesis (8.6%), E, Amino acid transport and metabolism (8.5%), and G, Carbohydrate transport and metabolism (8.1%), except for S, Function unknown (8.7%). The COG distribution of the accessory and unique genes was characterized by enrichment with those involved in information storage and processing, namely, L, Replication, recombination, and repair (20.4 and 23.8%, respectively), and K, Transcription (19.7 and 23.2%, respectively).

### Amino acid substitutions in PBPs and quinolone resistance-determining regions (QRDRs) of GyrA, GyrB, and ParC.

**FIG 3:**
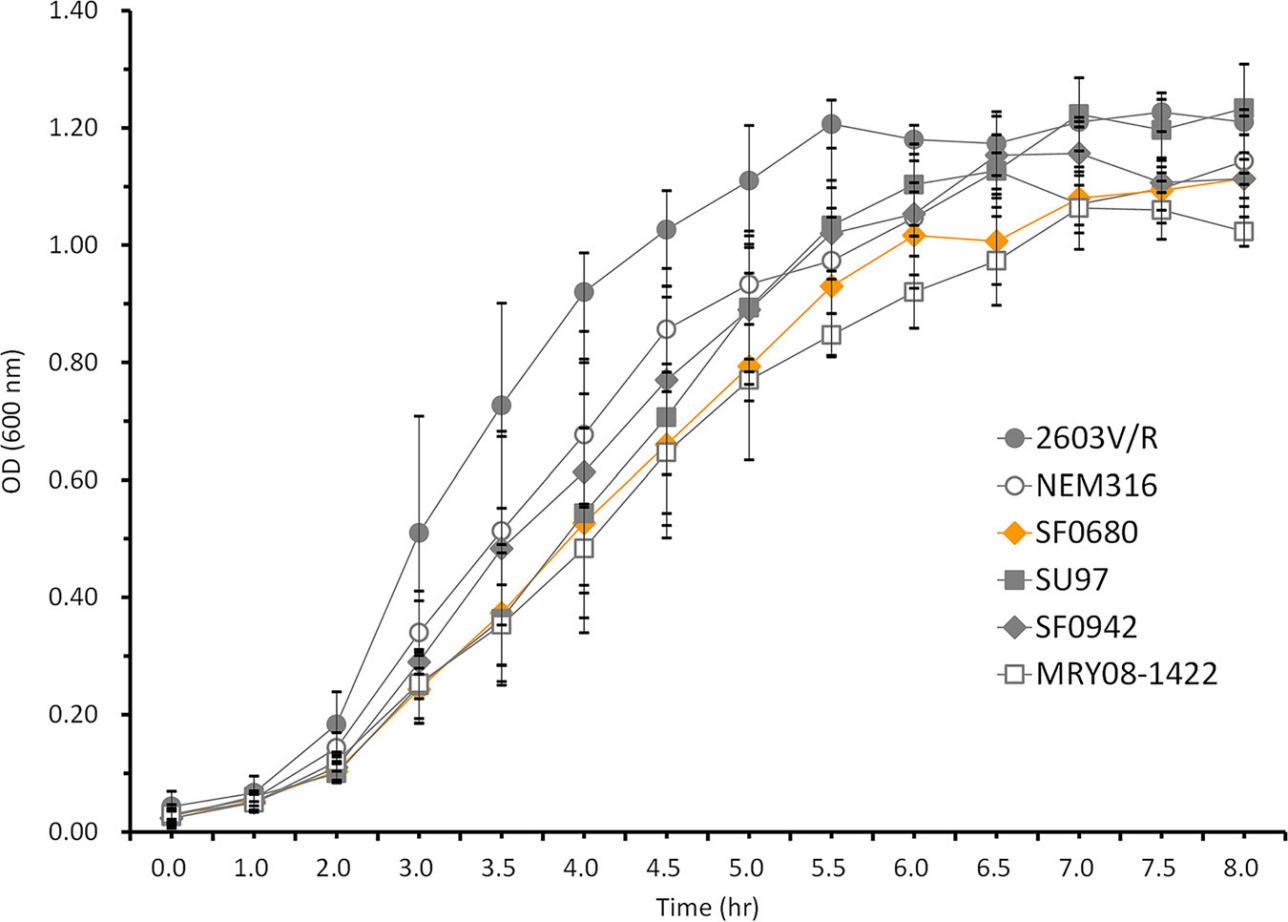
Comparison of growth curves of strain SF0680 harboring defective PBP2A and five strains with wild-type PBP2A, SU97, SF0942, MRY08-1422, 2603V/R, and NEM316. Data plotted are expressed as mean values ± standard deviations (SD).

Among the six ST1 PRGBS isolates with a penicillin MIC of 0.25 μg/mL (Table 1), serotype Ia/ST1 strains SU85, SU97, SU187, and SU233 shared the V405A mutation (a change of V to A at position 405) together with G329V, G398A, and G429D mutations in PBP2X and T587I in PBP1A, while serotype III/ST1 strains SF0942 and SF1510 shared I377V, F395L, R433H, H438Y, V510I, and G648A in addition to V405A in PBP2X and T567I in PBP2B. The serotype III/ST464 (CC23) strain MRY08-1422 with a penicillin MIC of 0.25 μg/mL had substitutions I377V, G398A, Q412L, and H438Y in PBP2X and F524V and G719N in PBP1A. The remaining serotype Ib/ST10 strain, SF0680, exhibiting a higher penicillin MIC of 1 μg/mL, had substitutions V405A, Q557E, and G398A in PBP2X. Notably, a frameshift mutation involving a single nucleotide insertion (T) at position 567, resulting in a premature stop codon, was identified in the *pbp2a* gene of this strain. Comparative growth curve analysis was performed to investigate the impact of this disrupted PBP2A on the cellular growth of this strain. As shown by the results in Fig. 3, strain SF0680 displayed growth dynamics similar to those of the other PRGBS strains, SU97, SF0942, and MRY08-1422, as well as that of GBS strain NEM316.

Amino acid substitutions in the QRDRs of the DNA gyrase (GyrA) and topoisomerase IV (ParC) have mainly been associated with fluoroquinolone resistance of GBS. Two serotype Ia/ST1 PRGBS strains, SU85 and SU97, with a levofloxacin MIC of 2 μg/mL (Table 1) shared S79Y and D83G in ParC, while the remaining two serotype Ia/ST1 strains, SU187 and SU233, with levofloxacin MICs of >8 μg/mL, carried G79C and S81L in GyrA, in addition to the double ParC substitutions. Two serotype III/ST1 strains, SF0942 and SF1510, one serotype Ib/ST10 strain, SF0680, and one serotype III/ST464 (CC23) strain, MRY-08-1422, with levofloxacin MICs of >8 μg/mL had S81L in GyrA along with S79F in ParC. Strain SF0942 also carried D83N in ParC and E476K in GyrB.

### Phylogenetic positions of eight invasive PRGBS isolates and predicted recombination.

**FIG 4:**
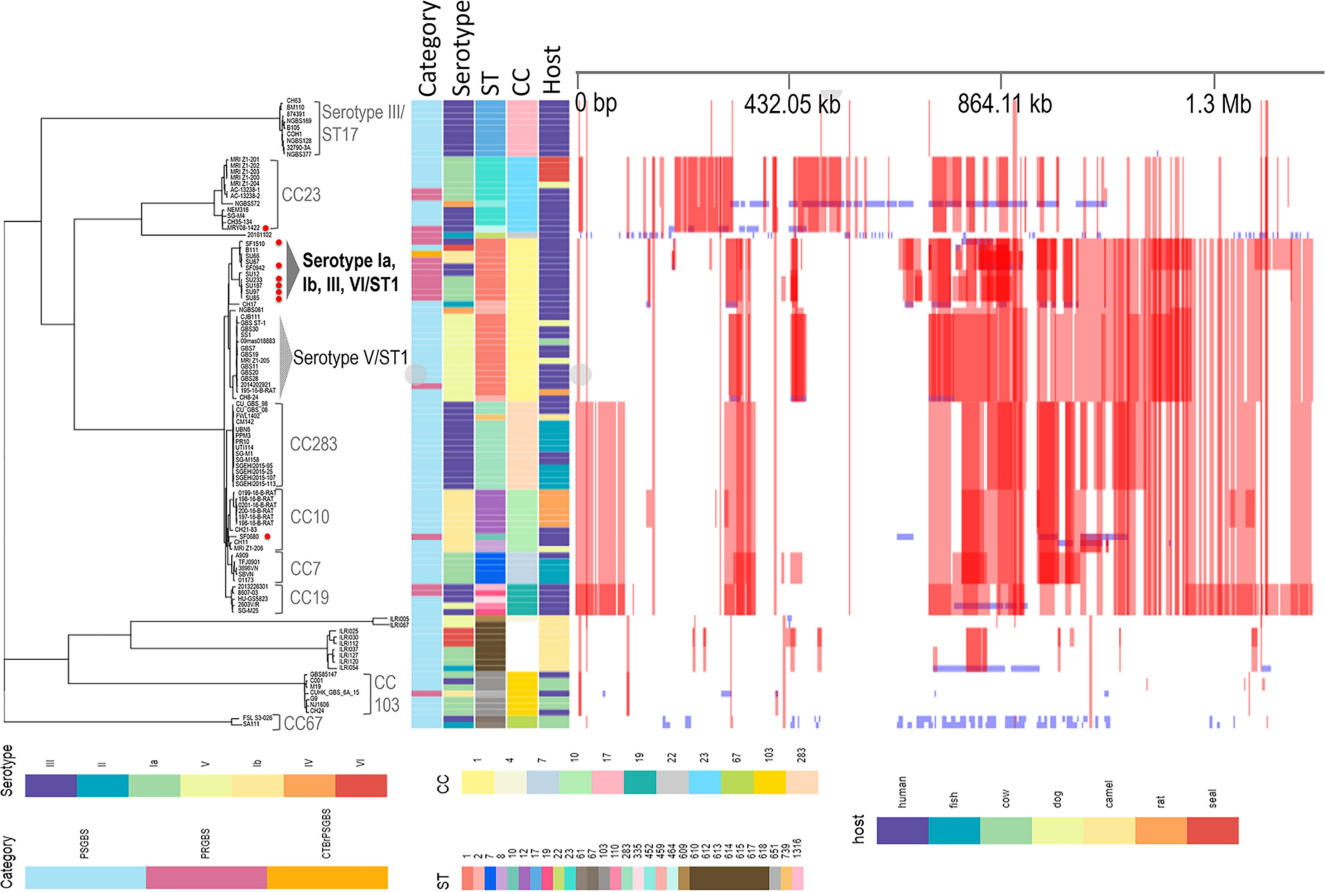
Phylogenomic analysis of 8 PRGBS isolates and 92 representative strains comprising 9 PRGBS, 1 CTB^r^PSGBS, and 82 PSGBS strains. Core genome SNP maximum-likelihood phylogenetic tree (left) was constructed from nonrecombinant regions of the whole-genome alignment of 100 genomes using Gubbins. The eight PRGBS isolates in this study are marked with red dots. Heat map (right) shows the densities of SNPs identified in genomic regions mapped to the reference genome of S. agalactiae 2603V/R. Red blocks and blue blocks indicate predicted recombinations occurring in the internal branches and in the terminal branches (unique to individual isolates), respectively.

The recombination-mutation ratio (*R*/*M*) per branch varied mostly depending on the ST (CC) to which the isolates belonged (Fig. 4). A relatively high *R*/*M* value of 9.67 was found for the clade of CC19 human isolates, while a low *R*/*M* value of 0.36 was estimated for the hypervirulent serotype III/ST17 clone associated with neonatal invasive infections. It is noteworthy that four serotype Ia strains, SU85, SU97, SU187, and SU233, and two serotype III strains, SF0942 and SF1510, of invasive PRGBS isolates in this study, together with other PRGBS/penicillin-susceptible GBS with reduced ceftibuten susceptibility (CTB^r^PSGBS) isolates mostly derived from the same hospital where the four serotype Ia isolates were obtained, formed an independent clade. The *R*/*M* value for the clade was found to be 3.97, which was 36 times higher than the *R*/*M* value of 0.11 for a serotype V/ST1 clade that mostly consisted of penicillin-susceptible GBS (PSGBS) isolates from human blood. The CC23 clade that predominantly consisted of serotype Ia and III isolates, including serotype III/ST464 PRGBS strain MRY08-1422, exhibited an *R*/*M* value of 0.88. An *R*/*M* value of 5.7 was estimated for the clade comprising subclades of CC283 that consisted predominantly of fish isolates, CC10 isolates including serotype Ib/ST10 PRGBS strain SF0680 and rat isolates, and CC7 isolates that consisted predominantly of fish isolates. Camel isolates were divided into two clusters, of which one consisted of seven isolates with an *R*/*M* value of 6.34 and the other of two isolates with *R*/*M* values of 0.03 to 0.06.

### Putative virulence and resistance gene profiles.

**FIG 5:**
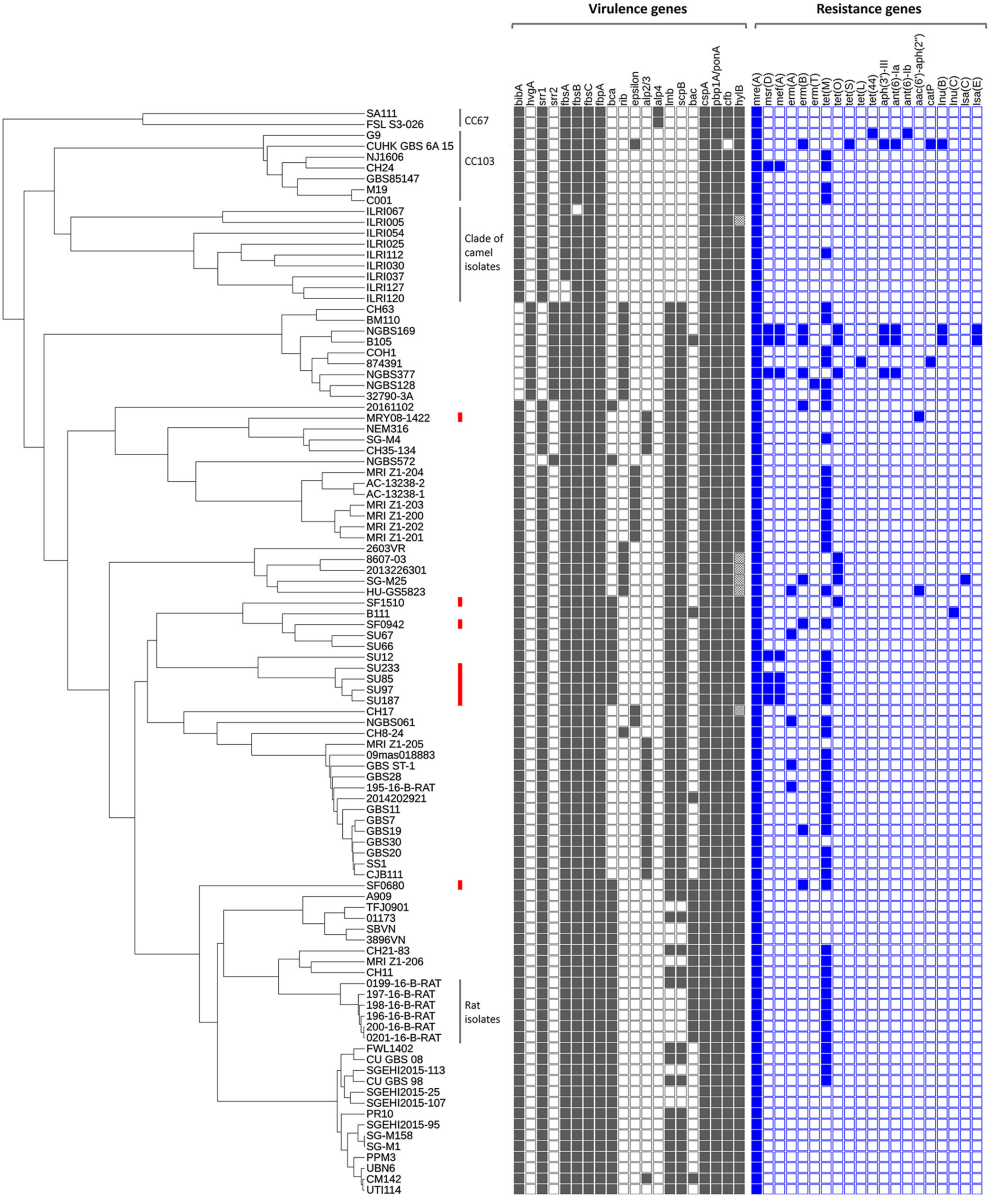
Whole-genome multilocus sequence typing (wgMLST) of 8 PRGBS isolates and 92 representative strains comprising 9 PRGBS, 1 CTB^r^PSGBS, and 82 PSGBS strains. The presence (filled squares) or absence (open squares) of virulence- and resistance-associated genes among strains is shown. A disrupted *hylB* gene is shown by a square filled with crosshatching. The eight PRGBS isolates in this study are marked with red bars.

A whole-genome multilocus sequence typing (wgMLST) tree of the 8 invasive PRGBS isolates and 92 reference strains for which genomes were available in NCBI (Table S1 in the supplemental material) and the repertoire of virulence- and resistance-associated genes detected in their genomes are shown in Fig. 5. The 92 strains were selected in an arbitrary manner from a wider GBS population that consisted of isolates from humans, animals, fish, etc. The wgMLST analysis revealed a separation of the clade that consisted mainly of PRGBS isolates, including all serotype Ia/ST1 and serotype III/ST1 invasive PRGBS isolates, from the clade that consisted solely of serotype V/ST1 isolates. The PRGBS isolates in this study shared most of the virulence genes, including *bibA*, *fbsA*/*-B*/*-C*, *cspA*, *cfb*, *hylB*, *scpB*, *lmb*, *srr1*, and the *cyl* operon, with other PRGBS/PSGBS isolates of human origin. However, disruption of the *hylB* structural gene was observed due to insertions of IS*1548* for four serotype III/CC19 isolates, including two PRGBS isolates of U.S. origin (8607-03 and 2013226301), IS*Sag4* for serotype II/ST2 strain CH17, and a Streptococcus phage Javan53 mobile element for serotype V/ST609 strain ILR1005. The absence of *lmb* and *scpB* genes in the CC103 and CC67 clades, which mostly consisted of cow isolates, serotype Ib/ST12 rat isolates from the CC10 clade, and a clade of camel isolates, was noted.

Six invasive PRGBS isolates, comprising four serotype Ia/ST1 PRGBS strains, SU85, SU97, SU187, and SU233, one serotype Ib/ST10 strain, SF0680, and one serotype III/ST1 strain, SF0942, harbored *tet*(M) (Fig. 5). The *tet*(M) gene was also detected in most of the reference isolates of human origin, whereas it was scarce among isolates from fish, camels, and cows. Strains SF0680 and SF0942 had *erm*(B), and strain SF1510 had *tet*(O). Three serotype Ia/ST1 PRGBS strains, SU85, SU97, and SU187, had *mef*(A)-*msr*(D), which was also detected in only five of the reference isolates of human origin. The aminoglycoside resistance gene *aac(6′)*-*aph(2*″) was detected in serotype III/ST464 PRGBS strain MRY08-1422 and one of the reference isolates, strain HU-GS5823. However, a 4.5-kb fragment containing *aac(6′)*-*aph(2″)* surrounded by IS*256* elements, the nucleotide sequence of which was identical to that of *staphylococcal* transposon Tn*4001*, was identified only in strain MRY08-1422 (35). The *mre*(A) gene was found in all PRGBS/PSGBS isolates analyzed. The *mre*(A) gene, encoding a flavokinase, was originally reported as a macrolide efflux gene in S. agalactiae (36). However, the gene has been detected in GBS isolates irrespective of erythromycin susceptibility, suggesting its ubiquitousness in this bacterial species (37).

In addition, manual exploration of the Roary gene_presence_absence.csv file and Microbial Nucleotide BLAST analysis found a few putative virulence genes among four invasive PRGBS isolates of serotype Ia/ST1. Namely, *pezTA* toxin-antitoxin genes were identified within a truncated form of a Tn*5253*-like element. The 1,247-bp fragment containing *pezT and pezA* that was located upstream from *pezT* exhibited 97.8% and 93.7% nucleotide sequence identities with those of S. pneumoniae (strain 19F; CGWC01000036) and S. agalactiae (strain CCUG 38383; ALQS01000023), respectively. These four isolates also harbored CAAX protease and bacteriocin-processing (CPBP) family intramembrane metalloprotease genes in a 1,221-bp fragment that exhibited 99.9% nucleotide sequence identity with that of S. pneumoniae (strain BVJ1JL; NZ_CP071871). The BLAST analysis showed a low query coverage of approximately 44% (96% nucleotide sequence identity) for that fragment from S. agalactiae (strain CCUG 38383; ALQS01000023).

## DISCUSSION

In our previous study, a poor clinical outcome was observed in all four elderly patients who had serotype Ia/ST1 PRGBS isolated from blood cultures (26), prompting us to investigate antibiotic resistance and virulence traits of MDR PRGBS associated with bloodstream infection in adults.

This first genomic study of invasive PRGBS in Japan confirmed the close genomic relationship of four serotype Ia/ST1 PRGBS isolates of nosocomial origin. Three of these serotype Ia/ST1 strains, SU97, SU187, and SU233, were detected at 1, 14, and 20 months, respectively, after the isolation of SU85 (26). Our previous studies have found persistent survival of serotype Ia/ST1 PRGBS at the site of infection for more than 3 weeks (38). Thus, the presence and persistence of such PRGBS isolates may increase the risk of bloodstream infections in elderly adults.

The pangenome of GBS has consistently been found to be open, with the pangenome curve showing no evidence of plateauing (39, 40). The open nature of the pangenome can be observed in many clinically relevant bacterial species that can inhabit multiple environments and undergo frequent gene gains by horizontal gene transfer, contributing to their genomic evolution (41). In this study, the pangenome of eight invasive PRGBS isolates is also suggested to be still open. However, the pangenome curve may almost reach a plateau with the exponent parameter value of 0.210, meaning the pangenome is probably going to be closed soon. The inconsistency regarding the openness (GBS) and almost closed status (PRGBS) of the pangenome might be due to the small number of genomes analyzed (42). The COG functional analysis revealed that accessory/unique genes within the eight invasive PRGBS isolates were enriched for the COG functional categories L, Replication, recombination, and repair, including genes involved in horizontal gene transfer events, and K, Transcription, findings that are similar to previous descriptions of GBS isolates (43). The increase in the frequencies of these COG L and K functional gene categories would facilitate the adaptation of the organism to altered environments or new niches (44, 45).

In Gubbins-based recombinational analysis, the maximum-likelihood (ML) phylogenomic tree of 100 genomes showed concordance with their ST (CC) distribution. Notably, a clade comprising our six invasive PRGBS isolates of serotypes Ia and III/ST1 and four ST1 PRGBS/CTB^r^PSGBS reference isolates mostly derived from the same hospital where the serotype Ia isolates were obtained was separated from a clade of serotype V/ST1 reference isolates mostly derived from human invasive infections. It was found that genetic recombination occurred more frequently within the former clade than in the latter clade. The increasing impact of invasive ST1 GBS infections, predominantly associated with serotype V, in nonpregnant adults has been recognized. Genomic evolution of these serotype V/ST1 isolates by converting to serotypes Ib, II, and IV through large-scale recombinations (46) is observed, and the acquisition of AlpST-1, a predicted surface-exposed adhesin protein, by ST1 isolates is noted (11). ST1 has been the predominant sequence type among PRGBS isolates in Japan (26, 31), and serotypes Ia and III have been very rare among ST1 GBS isolates (2, 47). Therefore, lineages dominated by serotype Ia and III/ST1 invasive PRGBS isolates could possibly emerge through recombination within the ST1 GBS populations (26, 40, 46). The wgMLST clusters generated based on loci and their sequence variations in the pangenome were highly concordant with the core single-nucleotide polymorphism (SNP)-based clusters, supporting the distinction of these ST1 clusters.

All eight invasive PRGBS isolates retained a core virulence gene repertoire (*bibA*, *fbsA*/*-B*/*-C*, *cspA*, *cfb*, *hylB*, *scpB*, *lmb*, and the *cyl* operon), supporting an invasive ability of our PRGBS isolates comparable to those of other human invasive PSGBS and noninvasive PRGBS isolates. Additionally, the presence of the AlpST-1 virulence gene was confirmed in four serotype Ia/ST1 PRGBS isolates, which may provide an additional benefit for their colonization (11).

Amino acid substitutions described previously in PBP2X, PBP2B, and PBP1A were identified in eight invasive PRGBS isolates with penicillin MICs of 0.25 to 1 μg/mL (nonsusceptible interpretive category) (21–23, 26, 38). Moreover, serotype Ib/ST10 PRGBS strain SF0680 had a disrupted PBP2A due to a frameshift mutation in the transglycosylase region. Strain SF0680 was very similar to other PRGBS and strain NEM316 in its growth dynamics, suggesting that the disrupted PBP2A did not affect the strain’s ability to grow, as has been described previously (48, 49).

The eight invasive PRGBS isolates harbored different sets of antimicrobial resistance genes according to their phenotypic resistance profiles. Namely, the four serotype Ia/ST1 PRGBS strains, SU85, SU97, SU187, and SU233, exhibiting resistance to minocycline, harbored *tet*(M), and the three of those strains that were also resistant to erythromycin harbored *mef*(A)-*msr*(D). The acquisition of *mef*(A)-*msr*(D) by GBS/PRGBS is rare, but three serotype III/ST17 GBS isolates from the blood of neonates possessing *mef*(A)-*msr*(D) were noted in our analysis. The serotype Ib/ST10 strain SF0680 and serotype III/ST1 strain SF0942, which exhibited resistance to erythromycin, clindamycin, and minocycline, had *erm*(B) and *tet*(M) genes, while the serotype III/ST1 strain SF1510, which was resistant to minocycline, had the bovine-associated *tet*(O) gene. Of note, the presence of the *aac(6′)*-*aph(2″)* gene, encoding a bifunctional enzyme conferring high-level resistance to gentamicin, was first confirmed in Japan in an invasive PRGBS isolate (serotype III/ST464 strain MRY08-1422) exhibiting high-level aminoglycoside resistance (gentamicin MIC of >1,024 μg/mL). The *aac(6′)*-*aph(2″)* gene is prevalent in enterococci and staphylococci, while it is still uncommon in GBS, representing only 0.27% of 6,340 isolates recovered during 2015 to 2017 through population-based Active Bacterial Core surveillance (ABCs) in the United States (47). This study revealed that PRGBS isolates could acquire Tn*4001* carrying *aac(6′)*-*aph(2″)*, so a risk may lie in the spread of this resistance gene among GBS/PRGBS as a vector.

The genomic analyses enabled us to detect some other genes that encode putative virulence factors in serotype Ia/ST1 PRGBS isolates. Type II toxin-antitoxin genes *pezT* and *pezA* were found within a putative Tn*5253*-like element. Activation of PezT through proteolytic degradation of PezA during environmental stress or bacterial infections would trigger the lysis of bacterial cells, resulting in the release of virulence factors that facilitate the expansion of infectious lesions (50). Also, the PezT toxin itself could possibly be lethal to the infected host cells (51). The CPBP family intramembrane metalloprotease contains YdiL (CAAX protease) and Abi (CAAX protease self-immunity) domains. The abortive infection (Abi) gene was found to be located immediately downstream from the Tn*5253* bacteriocin gene, and the involvement of the Abi gene in immunity to the cognate bacteriocin in Gram-positive bacteria has been described (52).

In conclusion, this study has shown that major virulence genes of invasive GBS could be identified in MDR PRGBS blood isolates from elderly patients in Japan. Moreover, the emergence of evolutional lineages of invasive PRGBS isolates of serotype Ia/ST1 and serotype III/ST1 that were distinct from the serotype V/ST1 PSGBS lineage consisting mostly of human blood isolates is confirmed. Among some invasive PRGBS isolates, the acquisition of mobile genetic elements associated with the antibiotic resistance gene *mef*(A)-*msr*(D) or *aac(6′)*-*aph(2″)* was noted. There is a trend toward increasing occurrence of penicillin nonsusceptibility among GBS isolates from various clinical sources, from 4.8% (472/9,738 isolates) in 2008 to 5.8% (2,355/40,258 isolates) in 2020 according to the Japan Nosocomial Infections Surveillance (JANIS) data of the Ministry of Health, Labor and Welfare (https://janis.mhlw.go.jp/english/index.asp). In the U.S. Active Bacterial Core surveillance (ABCs) of invasive GBS isolates from nonpregnant adults, the frequency of isolates with elevated MICs or *pbp2x* variants suggestive of β-lactam nonsusceptibility increased from 0.7% (13/1,835 isolates) in 2015 to 1.0% (20/1,953 isolates) in 2016 (1). In adults, the mortality rate was significantly higher among patients with bacteremia than among those without bacteremia (53). Our study highlights the need for large population-based genomic surveillance studies to better understand the clinical relevance of invasive PRGBS isolates.

## MATERIALS AND METHODS

### Bacterial strains.

A total of eight PRGBS clinical strains of blood origin with penicillin MICs of ≥0.25 μg/mL were recovered from elderly patients between 2008 and 2016 in Japan, all of which were subjected to our analyses; they included serotype Ia/ST1 strains SU85, SU97, SU187, and SU233, associated with nosocomial infections in a regional general hospital (26), and serotype III/ST464 (CC23) strain MRY08-1422 (31), serotype III/ST1 strains SF0942 and SF1510, and serotype Ib/ST10 strain SF0680 from sporadic cases of infection in a large-scale hospital. The GBS strains were grown in Todd-Hewitt broth (THB; BBL Microbiology Systems, Cockeysville, MD) and were then stored in glycerol at −80°C until use. Capsular serotypes were determined using antisera (Denka Seiken, Tokyo, Japan).

For growth curve experiments, overnight cultures were inoculated 1:100 into 50 mL of THB and the cells were incubated aerobically with agitation at 37°C. Growth was monitored over time by measuring the optical density at 600 nm (OD_600_) using a Biowave CO8000 cell density meter (Biochrom, Cambourne, United Kingdom). All experiments were performed three times independently.

### Antimicrobial susceptibility testing.

MICs were determined by the broth microdilution method using the MicroScan MICroFAST panel type 5J (Beckman Coulter, Tokyo, Japan). Alternatively, broth microdilution panels prepared in-house were used to provide a broader range of antimicrobial concentrations for evaluation of the MICs of ciprofloxacin (Tokyo Kasei Kogyo, Tokyo, Japan) and gentamicin (Fujifilm Wako Pure Chemical Co., Osaka, Japan) (54). The susceptibility categories were interpreted according to Clinical and Laboratory Standards Institute (CLSI) guidelines (55). S. pneumoniae ATCC 49619 was used as a quality control strain.

### WGS.

Whole-genome sequencing (WGS) and *de novo* assembly were conducted as described previously (26). Briefly, the genome was sequenced using the 150-bp paired-end method on the NovaSeq6000 platform system (Illumina, San Diego, CA), and the resulting raw reads were assembled *de novo* using SPAdes version 3.14.1. Molecular serotypes and STs were confirmed *in silico* using the genomic sequences according to previously described methods (26). The overall similarity between genome sequences was determined using OrthoANI version 0.93.1 (https://www.ezbiocloud.net/tools/orthoani) (56).

### Prediction of antimicrobial resistance genes, virulence genes, and ICEs.

Putative T4SS-type ICEs carrying genes encoding mobilizable elements (*oriT*, relaxase, and type IV coupling protein [T4CP]) and mating-pair formation elements (Tra and VirB4) were detected by ICEfinder (https://bioinfo-mml.sjtu.edu.cn/ICEfinder/index.php). Virulence genes and antimicrobial resistance genes in the putative ICEs, assembled genomes of 8 PRGBS, and 92 reference genomes available from the NCBI, comprising 2 PRGBS and 1 CTB^r^PSGBS isolate in Japan, 7 PRGBS isolates from other countries, and 82 PSGBS isolates (Table S1), were analyzed using VFAnalyzer from the virulence factors database (VFDB; http://www.mgc.ac.cn/VFs/), the comprehensive antibiotic resistance database (CARD; https://card.mcmaster.ca/), ResFinder 4.1 from the Center for Genomic Epidemiology (http://www.genomicepidemiology.org), and the BLASTn tool. Manual verification of the nucleotide sequences of those predicted virulence genes and resistance genes was performed on WGS data.

### Pangenome analysis.

The *de novo-*assembled genomes were annotated with Prokka (Galaxy version 1.14.5), producing output gff3 files that were used for pan- and core genome analyses using Roary (Galaxy version 3.13.0) on a Galaxy-based platform. An ML phylogenetic tree based on the core genome alignment was generated with RAxML in Galaxy. The Roary gene_presence_absence.csv file and the core genome tree were visualized using Phandango (57). BPGA pipeline version 1.3 was used to perform COG annotation to assign COG functional categories to the genes (58). Manual exploration of putative virulence-associated genes was also performed based on the Roary gene_presence_absence.csv file generated from the 100 genomes, including the 92 reference genomes (Table S1), and Microbial Nucleotide BLAST analysis.

### Recombination analysis.

A single-nucleotide polymorphism (SNP)-based maximum-likelihood (ML) phylogenomic tree and SNP densities used to predict recombination and horizontal gene transfer events were generated across the genomes of 100 isolates comprising the 8 invasive PRGBS isolates in this study and 92 reference strains by using Gubbins (Table S1). Namely, the core genome SNPs based on whole-genome alignments of the 100 genomes were generated using Parsnp (version 1.5.6) implemented in the Harvest suite (59). S. agalactiae 2603V/R (GenBank NC_004116) was used as the reference. The resulting alignment in XMFA format, which was converted to FASTA format, was then used as the input for Gubbins (Galaxy version 0.1.0) to perform recombination analysis and filter SNPs within recombinant regions in the core SNP alignment. The resulting tree and recombination events were visualized using Phandango (57).

### wgMLST analysis.

A whole-genome multilocus sequence type (wgMLST) tree of the 100 genomes was constructed based on alleles of all genes in the pangenome using PGAdb-builder (http://wgmlstdb.imst.nsysu.edu.tw) (Table S1). The phylogenetic tree was visualized using Interactive Tree of Life (iTOL) version 5 (http://itol.embl.de/).

### Data availability.

Genome assemblies for the eight PRGBS strains, SU85, SU97, SU187, SU233, SF0680, SF0942, SF1510, and MRY08-1422, have been deposited in the DDBJ/EMBL/GenBank database under GenBank assembly accession numbers GCA_020111495.1, GCA_020111445.1, GCA_020111535.1, GCA_020111505.1, GCA_020111555.1, GCA_020111435.1, GCA_020111455.1, and GCA_020111565.1 and BioProject accession number PRJNA649379.
