# Supplementary material for: Genomic Traits Associated with Virulence and Antimicrobial Resistance of Invasive Group B Streptococcus Isolates with Reduced Penicillin Susceptibility from Elderly Adults
Source: Microbiol Spectr. 2022 May 31;10(3):e00568-22. doi: 10.1128/spectrum.00568-22 (PMC9241772; doi:10.1128/spectrum.00568-22)
Supplement: Supplemental file 1 — Supplemental material. Download spectrum.00568-22-s0001.pdf, PDF file, 0.2 MB [file spectrum.00568-22-s0001.pdf]

TABLES1 List of 92 PSGBS/PRGBS reference genomes

Category	Strain	NCBI accession number	Sequence type	Clonal complex	Sero-type	Host species	Source	Country
PRGBS	20161102	SAMN06032651	22	22	II	human	(invasive)	United States
	2013226301	SAMN06032015	1316	19	III	human	(invasive)	United States
	8607-03	SAMN06032042	19	19	III	human (elderly)	blood	United States
	2014202921	SAMN06335547	1	1	V	human	sterile site	United States
	CUHK_GBS_6A_15	SAMN14145430	651	103	Ib	human (elderly)	infection on left foot	China
	SU12	SAMN26811719	1	1	III	human (elderly)	transtracheal aspirates	Japan
	SU67	SAMN26811721	1	1	Ib	human (elderly)	transtracheal aspirates	Japan
	AC-13238-1	SAMEA5842699	23	23	Ia	human (elderly)	drainage fluid (abscess in the right upper leg)	Germany
	AC-13238-2	SAMEA5842700	23	23	Ia			
CTB'PSGBS	SU66	SAMN26811720	1	1	Ib	human (elderly)	transtracheal aspirates	Japan
PSGBS	2603V/R	NC_004116.1	110	19	V	human	(invasive)	
	A909	NC_007432.1	7	7	Ia	human (neonate)	blood	
	BM110	NZ_LT714196.1	17	17	III	human (neonate)	(invasive)	United States
	COH1	NZ_HG939456.1	17	17	III	human (neonate)	blood	United States
	NEM316	NC_004368.1	23	23	III	human (neonate)	blood	
	CU_GBS_08	NZ_CP010874.1	283	283	III	human	blood	China
	CU_GBS_98	NZ_CP010875.1	283	283	III	human	cerebrospinal fluid	China
	SG-M1	NZ_CP012419.2	283	283	III	human	blood	Singapore
	SG-M158	NZ_CP021864.1	283	283	III	human	blood	Singapore
	SG-M25	NZ_CP021867.1	19	19	III	human	blood	Singapore
	SG-M4	NZ_CP021870.1	23	23	III	human	blood	Singapore
	32790-3A	NZ_CP029561.1/	17	17	III	human (neonate)	blood	China
	874391	NZ_CP022537.1	17	17	III	human	vagina	Japan
	SGEHI2015-25	NZ_CP025029.1	283	283	III	fish (bighead carp)	muscle	Singapore
	SGEHI2015-113	NZ_CP025026.1	283	283	III	fish (tilapia)	muscle	Singapore
	SGEHI2015-107	NZ_CP025027.1	283	283	III	fish (snakehead)	muscle (fresh fish)	Singapore
	SGEHI2015-95	NZ_CP025028.1	283	283	III	fish (silver carp)	muscle (fresh fish)	Singapore
	NGBS128	NZ_CP012480.1	17	17	III	human (neonate)	blood	Canada
	NGBS061	NZ_CP007631.1	459	1	IV	human	blood	Canada
	NGBS572	NZ_CP007632.1	452	23	IV	human	Synovial fluid	Canada
	HU-GS5823	NZ_AP018935.1	335	19	III	human	blood	Japan
	SS1	NZ_CP010867.1	1	1	V	human	blood	United States
	GBS30	NZ_CP042002.1	1	1	V	human	blood	United States
	GBS28	NZ_CP042001.1	1	1	V	human	blood	United States
	GBS20	SAMN12355373	1	1	V	human	blood	United States
	GBS19	NZ_CP042000.1	1	1	V	human	blood	United States
	GBS11	NZ_CP041999.1	1	1	V	human	blood	United States
	GBS7	NZ_CP041998.1	1	1	V	human	blood	United States
	CJB111	NZ_CP063198.2	1	1	V	human (neonate)	blood	United States
	GBS85147	NZ_CP010319.1	103	103	Ia	human	oropharynx	Brazil
	C001	NZ_CP008813.1	103	103	III	cow	milk	China
	NJ1606	NZ_CP026084.1	103	103	Ia	cow	milk	China
	SA111	NZ_LT545678.1	61	67	II	bovine	milk	Portugal
	FSL S3-026	SAMN02428925	67	67	III	bovine	milk	United States
	GBS ST-1	NZ_CP013202.1	1	1	V	dog	gumline	United States
	09mas018883	NC_021485.1	1	1	V	cow	milk	Sweden
	FWL1402	NZ_CP016391.1	739	283	III	frog		China
	ILRI005	NC_021486.1	609		V	camel	abscess	Kenya
	ILRI112	HF952106.1	617		VI	camel	abscess	Kenya

01173	NZ_CP053027.1	7	7	Ia	fish (seabass)	tissue	Kuwait
TFJ0901	NZ_CP034315.1	7	7	Ia	fish (tilapia)		China
0199-16-B-RAT	SAMN05762134	12	10	Ib	rat	nares	United States
0201-16-B-RAT	SAMN05762136	12	10	Ib	rat	Fetus	United States
195-16-B-RAT	SAMN05762126	1	1	V	rat	nares	United States
196-16-B-RAT	SAMN05762127	12	10	Ib	rat	Cardiac lesion	United States
197-16-B-RAT	SAMN05762129	12	10	Ib	rat	blood	United States
198-16-B-RAT	SAMN05762133	12	10	Ib	rat	nares	United States
200-16-B-RAT	SAMN05762135	12	10	Ib	rat	Cardiac lesion	United States
3896VN	SAMN09005654	7	7	Ia	fish	tissue	Viet Nam
B105	SAMN07178057	17	17	III	human (neonate)	blood	China
B111	SAMN07178058	1	1	VI	human (neonate)	blood	China
CH11	SAMN10055700	8	10	Ib	human	Vaginal-rectal	Kenya
CH17	SAMN10055702	2	1	II	human	Vaginal-rectal	Kenya
CH21-83	SAMN10055703	12	10	Ib	human	Pus	Kenya
CH24	SAMN10055704	103	103	Ia	human	blood	Kenya
CH35-134	SAMN10055705	23	23	III	human	urine	Kenya
CH63	SAMN10055706	17	17	III	human	Vaginal-rectal	Kenya
CH8-24	SAMN10055699	2	1	V	human	Vaginal-rectal	Kenya
CM142	SAMN08517483	283	283	III	fish	whole organism	Thailand
G9	SAMN20953728	103	103	Ia	bovine	-	China
ILRI025	SAMN06707234	610		VI	camel	milk	Kenya
ILRI030	SAMN06707235	617		VI	camel	milk	Kenya
ILRI037	SAMN06707236	612		Ia	camel	Gingivitis	Kenya
ILRI054	SAMN06707237	615		II	camel	Wound	Kenya
ILRI067	SAMN06707238	614		V	camel	swab	Kenya
ILRI120	SAMN06707239	618		Ia	camel	nasal	Kenya
ILRI127	SAMN06707240	613		Ia	camel	Pus	Kenya
M19	SAMN03292190	103	103	Ia	bovine	milk	China
MRI Z1-200	SAMN02436213	23	23	Ia	seal	-	United States
MRI Z1-201	SAMN02436251	23	23	Ia	seal	-	United States
MRI Z1-202	SAMN02436191	23	23	Ia	seal	-	United States
MRI Z1-203	SAMN02436258	23	23	Ia	seal	-	United States
MRI Z1-204	SAMN02436212	23	23	Ia	dog	-	United States
MRI Z1-205	SAMN02436306	1	1	V	dog	-	United States
MRI Z1-206	SAMN02436211	8	10	Ib	dog	-	United States
NGBS169	SAMN04093987	17	17	III	human (neonate)	blood, EOD	Canada
NGBS377	SAMN04094015	17	17	III	human (neonate)	blood, LOD	Canada
PPM3	SAMN08517486	283	283	III	fish	whole organism	Thailand
PR10	SAMN08517489	283	283	III	fish	whole organism	Thailand
SBVN	SAMN09005652	7	7	Ia	fish	tissue	Viet Nam
UBN6	SAMN08517520	283	283	III	fish	whole organism	Thailand
UTI114	SAMN08517522	283	283	III	fish	whole organism	Thailand

PRGBS, group B streptococci with reduced penicillin susceptibility; CTB^{r}PSGBS, penicillin-susceptible GBS with reduced cephalosporin susceptibility; PSGBS, penicillin-susceptible GBS.
